# Sub Maximal Ergospirometry Parameters in Untrained Non-Frail Octogenarian Subjects

**DOI:** 10.3390/medicina58030378

**Published:** 2022-03-03

**Authors:** Cristian Cofre-Bolados, Gerson Ferrari, Pedro Valdivia-Moral, Félix Vidal-Díaz, Robinson Ramírez-Vélez, Mikel Izquierdo-Redin

**Affiliations:** 1 Laboratory of Sciences of Physical Activity, Sport and Health, Faculty of Medical Sciences, Universidad de Santiago de Chile, Santiago 9170022, Chile; gerson.demoraes@usach.cl; 2 Human Performance Lab, Education, Physical Activity and Health Research Unit (GEEAFyS), Universidad Católica del Maule, Talca 3460000, Chile; 3 Faculty of Education, Department of Didactics of Musical, Plastic and Body Expression, University of Granada, 18071 Granada, Spain; pvaldivia@ugr.es; 4 Navarrabiomed, Hospital Universitario de Navarra (HUN), Navarra Institute for Health Research (IdiSNA), Universidad Pública de Navarra (UPNA), 31008 Pamplona, Navarra, Spain; felixvidaldiaz@gmail.com (F.V.-D.); robinson.ramirez@unavarra.es (R.R.-V.); mikel.izquierdo@unavarra.es (M.I.-R.); 5 CIBER of Frailty and Healthy Aging (CIBERFES), Instituto de Salud Carlos III, 28029 Madrid, Spain; 6 Facultad de Ciencias de la Educación, Unidad Central del Valle del Cauca, Tuluá 763022, Valle del Cauca, Colombia

**Keywords:** ergometry, cardiorespiratory risk, older adults

## Abstract

*Background and Objectives*: The prevalence of chronic diseases increases with age, and in octogenarian elderly, a cardiorespiratory test with gas analysis is more effective in determining the risk of mortality than applying the conventional risk factors. *Materials and Methods*: 25 untrained non-frail octogenarian subjects (four men) performed a submaximal test with gas analysis, which was stopped after the second ventilatory threshold (VT2) was reached. The variables analyzed were oxygen consumption at the first threshold (VO_2_ VT1); ventilatory class (VE/VCO_2_); oxygen uptake efficiency slope (OUES); cardiorespiratory optimal point (COP); oxygen pulse difference between VT2 and VT1 (diff. VO_2_/HR VT2-VT1). *Results*: the variables were classified categorically based on cut-off points present in the literature, where the variable with the highest percentage of altered cases was dif. VO2/HR VT2-VT1 at 48%; followed by VO_2_ VT1 at 40%, OUES at 36%, COP at 32%, and VE/VCO_2_ at 24%. Chi-square analysis between the measured parameters defined that normal and altered variables were related to each other, except for the variable VE/VCO_2_ and OUES. *Conclusions*: it was found that the main altered variable was the oxygen pulse and the least altered variable was VCO_2_/VCO_2_; there was only a statistically significant difference in a pair of OUES vs. VE/VCO_2_ variables.

## 1. Introduction

In Chile and in the world at large, we are in the midst of an “epidemiological transition”, characterized by reduced premature mortality, increased life expectancy, and a progressive reduction in birth rates [1]. According to the Chilean Department of Health Statistics and Information (DEIS), in 2002 the octogenarian population accounted for 1.41% of the national population, and for 2.5% of the population a decade later [2]. The prevalence of chronic diseases is also on the rise. The last National Health Survey in Chile (2009–2010) found that 74.4% of the population aged ≥65 years had high blood pressure, 41.8% were at high or very high cardiovascular risk, and 42.7% suffered from dyslipidemia. Polypharmacy is also common among the elderly, who consume an average of 4.2 drugs a day [3].

This ageing population calls for the implementation of elderly exercise programs to be recognized at global level. In turn, it poses new challenges for the physiology and methodology of health-oriented training, which now requires greater accuracy in the evaluation and prescription of exercise. Moreover, these challenges have been exacerbated by increased sedentary lifestyles in the severe acute respiratory syndrome, beta coronavirus (SARS-CoV-2) pandemic, which has limited mobility and access to physical activity in the elderly population.

Cardiopulmonary testing with gas analysis (CPX) is considered the standard criterion for assessing aerobic strength and its relationship to health. However, maximum CPX evaluation with and without gas analysis can be extremely challenging and even risky in certain cohorts. Consequently, submaximal stress tests constitute a highly reliable and valid alternative method, with a good safety profile, especially in ageing populations [4,5,6].

As CPX tests usually take the maximum oxygen consumption or peak oxygen consumption (VO_2_ max/VO_2_ peak) as the golden standard, there are established and very recent proposals [7] to try to estimate this VO_2_ max from submaximal tests. While recognizing the role of the maximum or peak parameter [8], the present study places the attention on the submaximal values of the CPX test, presented by different studies where there is evidence of cut-off points of cardiopulmonary and metabolic alteration in the oxygen consumption in the first ventilatory threshold (<11 mL/kg-min) [9], Ventilatory Class (Classes III and IV for slope in degrees) [10], OUES (<1550 mL), Cardiorespiratory Optimal Point (over 30 L minute) and Oxygen pulse difference between second ventilatory threshold and first ventilatory threshold (mL/heartbeats). These investigations also explore their respective cut-off points in populations with and without cardiovascular disease, with advanced age as a common element. These studies link associated CPX results with health profiles and survival rates across different cohorts, particularly in older adults. 

A recent study of the UK Biobank [4] of 58,892 adult and elderly participants determined that cardiorespiratory fitness measured by submaximal exercise testing was better at predicting mortality risk than conventional risk factors, and that its prognostic significance varies in people with cardiovascular risk levels. 

The aim of the study is to describe selected submaximal parameters of gas analysis ergometry in a sample of non-fragile and untrained octogenarian adults, who have spent 18 months without regular physical exercise due to the limitations of the SARS-CoV-2 pandemic, and to compare the results obtained against values proposed in other studies used as a reference for analysis.

## 2. Materials and Methods

### 2.1. Subjects

The sample size consisted of 25 subjects in their 80s (4 men), selected from physical activity programs for the elderly in the Young Men’s Christian Association (YMCA) in Santiago, Chile, and in the Adapted Exercise Center (CEA), all of whom had returned to their exercise programs after 18 months of inactivity linked to the SARS-CoV-2 pandemic conditions. All participants were non-frail and self-reliant, based on the Powerfrail application, and had a pre-pandemic history of physical exercise in YMCA programs in Santiago. During the period of confinement (considered detraining), none of them carried out scheduled or professionally led physical activity. The first activity they undertook before restarting their exercise programs was the submaximal CPX test.

All subjects gave their informed consent. The study protocol was approved by the University of Santiago Ethics Committee and complied with the principles of the Declaration of Helsinki.

### 2.2. Intervention Submaximal Ergometry Test with Gas Analysis

Study participants were subjected to a pre-participatory preventive medical review by physicians specializing in sports and physical activity medicine, where health parameters such as resting heart rate, arterial pressure, and general musculoskeletal status were measured. The purpose was to exclude possible underlying cases of cardiac, metabolic, and skeletal muscle diseases. In this case no participants were excluded.

**Figure 1:**
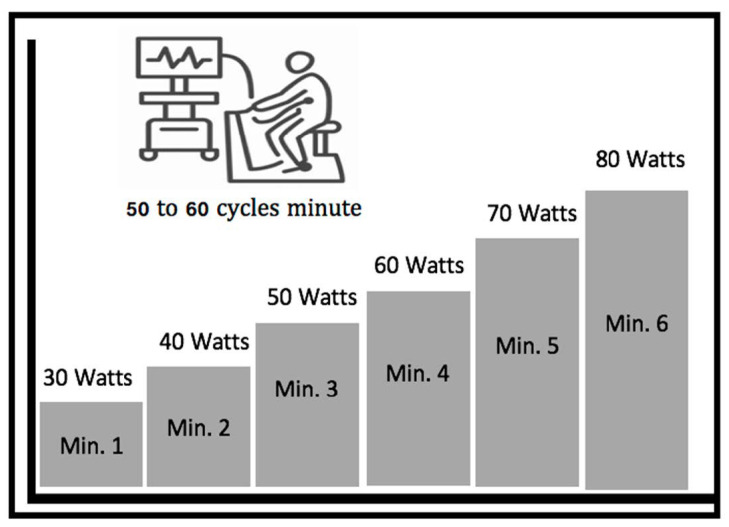
Ramp protocol with a 10 watts load increase per minute (aimed at elderly population with low aerobic power).

They were then subjected to a CPX submaximal test performed with the Cortex Metamax 3B gas analyzer, using an incremental protocol, adapted and executed on the Technogym Exite cycloergometer. The test started with an initial load (one) of 30 watts, which had increments of 10 watts each minute, maintaining a cadence of between 50 and 60 cycles per minute (Figure 1). The test was stopped after the second ventilatory threshold (VT2), determined according to Wasserman’s proposed graphs number six and nine (ventilatory equivalents and final expiratory pressure of oxygen and carbon dioxide, respectively) [8].

Once the test was stopped, the results were analyzed, and the values measured were categorized into five submaximal parameters. This allowed us to analyze the values obtained in this study and compare the results with normal cut-off points and altered parameters based on evidence on submaximal parameters obtained in CPX. The parameters have all been proposed in existing research:

(a) Oxygen consumption at first VT1 ventilatory threshold (VO_2_ VT1)

Expressed as the numeric value in milliliters (mL/kg-min) of oxygen (relative value) reached at the time the subject reaches the first ventilatory threshold (VT1), the cut-off for this parameter was 11 mL/kg, with <11 mL/kg-min classified as altered and >11 mL/kg-min as normal in relation to high risk of early death [9].

(b) Ventilator class (VE/VCO_2_)

This corresponds to the grade of the slope of VE/VCO_2_, classified in ventilatory classes I, II, III and IV and associated with cardiac event-free survival with better prognosis for classes I and II. It is considered an altered cut-off point when grades of this slope enter ventilatory class 3 and 4 (over 36°), according to the Arenas 2007 [10] studies and rating.

(c) Oxygen uptake efficiency slope (OUES or Log VE):

This represents the ratio of minute ventilation (VE) to oxygen absorption during gradual exercise. Cardiovascular, respiratory and musculoskeletal fitness are incorporated into this single index. The advantage of OUES is that it can be determined in a test that is interrupted before the end or in a submaximal protocol. OUES is highly correlated with other parameters such as peak VO_2_ and ventilatory thresholds. Higher OUES means higher aerobic capacity and strength, its cut-off point corresponds to <1550 as altered and >1550 as the normal value [11].

(d) Cardiorespiratory Optimal Point (COP)

This is a novel index, calculated as the minimum ventilatory equivalent of average oxygen (VE/VO_2_) over the lowest minute of this parameter. Its prognostic value has been demonstrated both independently and in combination with maximum oxygen consumption (VO_2_ max) in community-resident adults [12]. A recent preliminary report in Portugal [13] identified that COPs over 30 were associated with mortality in 487 patients with heart failure who were followed for an average time of 11 months, where the highest AUC value (0.915) among several other CPET variables, showed stronger prognostic strength than the VO_2_ peak.

(e) Oxygen pulse difference between VT2 and VT1(Dif. VO_2_/HR in VT2 vs. VT1):

During the exercise, this variable represents the increase in the systolic volume needed to increase the effort, reaching maximum values for oxygen consumption. When this parameter does not increase or even decreases with incremental effort, we are faced with an abnormal systolic volume response [14]. An oxygen pulse equal to or less in VT2 vs. VT1 is considered poor.

### 2.3. Statistics

Data distributions were analyzed using the Shapiro-Wilk test. Quantitative data were recorded as minimum, maximum, mean, and standard deviation. For categorical data, we used existing frequency and percentage. The frequency of normal and altered cases was calculated for each parameter and the variables were compared with their categorical results by Chi-square (X^2^), using an established significance level of *p* < 0.05. The statistical analyses were performed using the social science software package for Windows version 21.0 (IBM Corporation, New York, NY, USA).

## 3. Results

Table 1 shows descriptive results for the sample. Mean age, weight, height, and body mass index were 83.3 years, 66.5 kg, 158.4 cm and 25.9 kg/m^2^, respectively. Furthermore, 72% had a cardiometabolic disease and 64% used cardiometabolic drugs.

**Figure 2:**
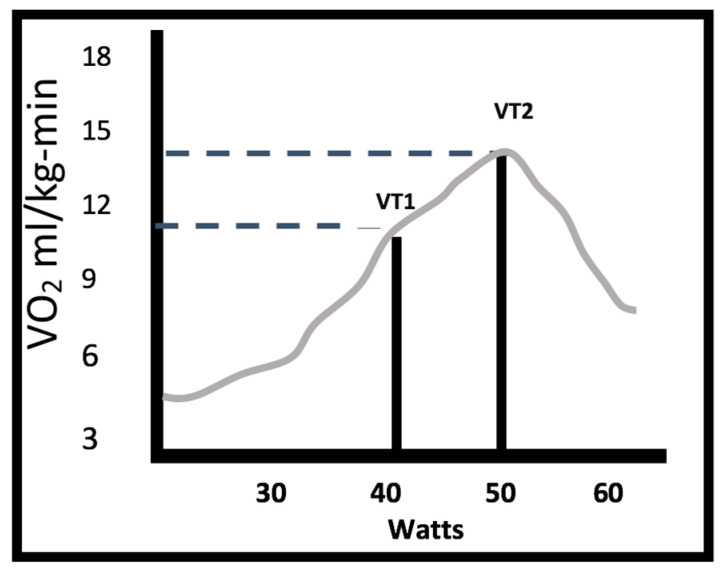
Watts and oxygen consumption (VO_2_) at first and second thresholds (VT1 with a VO_2_ of 11.08 mL/kg-min and 40 watts; VT2 with a VO_2_ of 13.9 mL/kg-min and 60 watts).

The results obtained from the analysis of CPX by analyzing the graphs proposed by Wasserman (Figure 2 for determining VO_2_/HR in VT1 and VT2; Graph 3 for VO_2_ in VT1 and OUES; Graph 4 for VE/VO_2_ ventilatory class; Graph 6 for obtaining POPs) is presented below.

The submaximal CPX test in the study group showed a VO_2_ VT1 of 11.08 mL/kg-min (SD 2.0) with a power of 40 watts, and a VO_2_ VT2 of 13.9 mL/kg-min (SD 1.8).

The main CPX analysis is presented in Table 2, from which the five fundamental variables of this study are selected.

Based on a review of the specialized literature, five disseminated submaximal variables associated with postoperative response, survival and mortality were selected. These were based on established cut-off points in different cohorts including groups with existing pathologies in order to represent the normal or altered condition of cardiorespiratory parameters in octogenarians, by identifying the percentage level of alteration or normality. Of the total sample (*n* 25) only two subjects did not present altered parameters, therefore 92% of the sample presented altered parameters, which are broken down into number and percentage (Table 3).

The following process corresponds to a comparison between variables to guide a possible relationship between the submaximal variables selected and described by their prognostic and predictive character when associated with risk factors. Table 4 presents the Chi-square test comparing the cut-off points (normal versus altered) of the variables analyzed. The results show significant differences for VE/VCO_2_ and OUES. For the other variables there were no significant differences.

## 4. Discussion

The results obtained in this study and their contrast with the other referenced research leads us to a detailed discussion for each of the five parameters analyzed, plus an analysis of the values and the relationship between the variables. It is important to mention that each parameter and its referenced studies are associated with different groups and conditions. 

Cohorts with pathologies have been incorporated due to the existence of studies with normal and altered values of the submaximal parameters studied in this population. These cohorts gave us a reference to decide cut-off points for values in our sample study. 

It is essential to mention that the octogenarian population usually presents cardiometabolic alterations associated with ageing, such as arterial hypertension, metabolic alterations in glycemic control, general metabolic syndrome and decreased autonomic function as well as decreased respiratory and cardiac values. All these conditions are associated with the exercise recommendations that allow people to prevent and control these conditions.

### 4.1. VO_2_ at First Threshold (VO_2_ VT1)

Stevenson [15] reported that VO_2_ in VT1 can replace peak VO_2_ in its prognostic role from the Weber classification [16,17] with CPX used as a measure of presurgical condition in older adults. The post-surgical risk cut-off point was determined at <11 mL/kg-min, defined in the Older and Hall study [18], which determined the same prognostic strength level compared to peak VO_2_, in relation to long-term mortality. We found that this cut-off point provided greater prognostic strength to predict 6-month mortality, even after multivariate adjustment for age, sex, and LVEF. The results of our study corresponded to an average of 11.3 mL/kg-min, which gave an average value slightly above the defined cut-off point.

### 4.2. Ventilator Class (VE/VCO_2_)

The VE/VCO_2_ slope is calculated from the linear relationship between VE and VCO_2_ 1 min after the start of a ramp protocol, and at the respiratory compensation point (CPR) or VT2 [19]. The physiological basis of this slope calculation is the potential risk of some hyperventilation at the beginning of the exercise test and the rapid increase in slope after VT2, due to hyperventilation in response to the fall of PaCO_2_. Wasserman et al. [20] recommend that the slope VE/VCO_2_ be determined by including data from the beginning of the exercise and at the VT2. A multi-center team showed that the VE/VCO_2_ slope had a higher prognostic value when all exercise test data (i.e., from the start of the test) were used compared to the method that included only a portion of the stress test, as was performed in an existing study [21]. Although there are many definitions in the literature, Arena et al. [22] showed that the slope of VE/VCO_2_ retains prognostic significance regardless of the achievement of 25%, 50%, 75% or 100% of the total test time considering 100% peak VO_2_ with maximality criteria. About a quarter of the present study sample showed alteration on this parameter.

### 4.3. Oxygen Uptake Efficiency Slope (OUES or Log VE)

By increasing the load and effort in ergometry, the VE/VO_2_ ratio is linear only up to VT1, i.e., during a small part of the exercise. Therefore, the VE/VO_2_ slope can be calculated as a linear equation only at the beginning of the exercise, when the slope is more erratic and is affected by psychological effects associated with the stress of the test or the situation associated with the test. In the late 1990s, Baba et al. [23] suggested that linearity could be maintained by applying the regression logarithm assuming an exponential curve. While this problem and its mathematical analysis have many possible points to analyze and solve, the question of interest to the present study is: *Does the OUES differ from the ventilatory class (VE/VCO_2_)?*

In response, it is defined that VE/VCO_2_ is mainly pulmonary efficiency, whereas VE/VO_2_ is a combination of lung capacity, circulation, and resistance of capillaries to mitochondria to oxygen flow, i.e., total body efficiency, and not only peripheral efficiency [24]. From this response, we have incorporated OUES independently of VE/VCO_2_, obtaining a further sub-maximal variable for our study. More than a third of the sample presented this altered variable.

### 4.4. Cardiorespiratory Optimal Point (COP)

The Laukkanen study [14] evaluated the association of COPs with cardiovascular disease and all causes of mortality. COP values could improve the prediction of ECV mortality when incorporated into risk factor data in a study population. However, isolated COPs are not more accurate than cardiorespiratory fitness, although one previous study [25] found that a COP > 30, independently or in combination with a low VO_2_ max, would be a good predictor of all causes of mortality in adults living in the community (whether apparently healthy or chronically ill). COP is a submaximal prognostic index that is easy to obtain and adds to the CPX assessment, especially for adults who cannot or do not want to achieve maximum exercise, as was the case in this study. Thus, the COP index is the fourth submaximal parameter exposed and analyzed in the present study, where it was found that about one third of the sample showed alteration in this parameter.

### 4.5. Oxygen Pulse Difference between VT2 and VT1(Dif. VO_2_/HR VT2 vs. VT1)

The normal physiological response to progressive exercise is a continuously increasing O_2_ pulse, a linear increase in VO_2_ vs. stress intensity, and a linear increase in heart rate versus VO_2_ to peak values [26,27,28]. The development of myocardial ischemia during exercise may result in reduced systolic volume, resulting in a flattened O_2_ pulse curve. It has been suggested that a descending O_2_ curve can indicate the presence of exercise-induced myocardial ischemia, assessed by myocardial perfusion imaging [29,30]. An association between myocardial fibrosis and an abnormality in the O_2_ pulse has also been documented [31]. Given the possibility and presence of many of the pathologic conditions studied and their association with older populations, particularly octogenarians, the difference in the oxygen pulse in VT2 vs. VT1 has been used, considering that no difference in favor of the VT2 value or even a drop in the oxygen pulse in VT2 vs. VT1 is abnormal. This was present in about half of the sample, which suggests that this cardiac parameter would be the main alteration of the study sample.

Relational analysis between parameters enabled us to see whether, when compared to each other, the parameters were related. This showed if there was a relationship in the deterioration and the association between certain alterations. The only parameter with significant differences in their results and categorical score of altered and normal, was the ventilatory class VE/VCO_2_ vs. OUES. It is important to emphasize that in this same section, in the discussion concerning OUES, the difference recently analyzed in these two parameters was explained by the association of central vs. peripheral dependency, generating the need to evaluate both parameters. We believe that further research is required, with larger samples and in other larger age groups, to identify differentiation related to different age groups. 

The study of the five parameters in a submaximal CPX test has no precedents found in our search. For this reason, the search for comparable reference values has been associated with different cohorts, including the general population as well as the population with pathologies [11,12,18,22,30]. In addition, it should be noted that there are very limited studies in octogenarians. The values presented here and the comparison with cut-off points that include groups with complex cardiac pathologies, such as heart failure or ischemia, do not correspond to an involuntary methodological error. This is because their inclusion seeks to find evidence on the impact and deterioration of some of the cardiopulmonary and metabolic parameters in the subjects exposed to situation of detraining, in this case associated with confinement derived from SARS-CoV-2. This allows us to establish some altered values compared to the general population, but also to the population with cardiac pathologies. The aim is to open future research for submaximal parameters in CPX tests and their behavior in the aged subject with and without pathologies.

The presence of altered parameters in most of the subjects in the sample (only two subjects did not present altered parameters) indicates that the parameters studied manifest alterations in octogenarians; this situation may be associated with the age and/or lack of physical training that the analyzed group presented.

We understand as a weakness of the study the limitation in the number of men included, which prevents identifying sex differences. Further related to the limited subject numbers in the sample, from the statistical analysis for categorical data in the classification of alteration or normality, there were some groups with less than 4 subjects, which we believe has not altered the global results of the analysis.

## 5. Conclusions

Results have been defined for normality and alteration of five submaximal parameters obtained from the CPX test in octogenarians who were untrained after 18 months without regular physical activity, due to limitations inherent to the SARS-CoV-2 pandemic. Findings showed that the main altered variable was the oxygen pulse, and the least altered variable was the ventilatory class. In addition, analysis between the variables discovered that there was only a statistically significant difference in one pair of variables: ventilatory class vs. OUES; no other relationships were found.

## Figures and Tables

**Table 1:** Characteristics (*n* = 25) of the octogenarian sample.

Variable	*n* (%)	Minimum	Maximum	Average	Standard Deviation
Age (years)		80.0	92.0	83.3	3.4
Weight (kg)		49.0	89.0	66.5	13.4
Height (cm)		150.0	176.0	158.4	7.3
Body mass index (kg/m^2^)		20.0	33.0	25.9	6.9
Cardiometabolic disease (%)	18 (72.0)	---	---	---	---
Cardiometabolic drugs (%)	16 (64.0)	---	---	---	---

**Table 2:** Average result of the submaximal variables measured, with their mean and standard deviation.

Variable	Minimum	Maximum	Average	Standard Deviation
VO_2_ VT1 (mL/kg-min)	8	16	11.08	2.0
Power VT1 (watts)	30	50	40	5.3
VE VT1 (L/min)	13.4	45.7	25	6.8
VO_2_/HRVT1 (mL/heart beats)	5	13	8	2.1
VO_2_ VT2 (mL/kg-min)	11	18	13.9	1.8
Power VT2 (watts)	40	60	50	7.2
VE VT2 (L/min)	24.1	52	34	7.1
VO_2_/HR VT2 (mL/heart beats)	8	15	8	2.4
COP (average min L)	23.3	37.6	29	3.4
VE/VCO_2_ (slope in degrees)	26.4	42.3	33	4.3
OUES (mL)	1380	1720	1501	90.0

VO_2_ (VT1) (oxygen consumption at first ventilatory threshold); VE (VT1) ventilation at first ventilatory threshold; VO_2_/HR (VT1) (Oxygen Pulse at first ventilatory threshold); VO_2_ (VT2) (oxygen consumption at second ventilatory threshold); VE (VT2) ventilation at second ventilatory threshold; VO_2_/HR (VT2) (Respiratory Point; Oxygen at second expiratory respiratory threshold VE/VCO_2_ (ventilatory class, ventilation divided by carbon dioxide volume); OUES (Oxygen uptake efficiency slope).

**Table 3:** Sub-maximum variables were “altered” when under the cut-off point for variables.

Variable	Cut-Off Point	Normal *n* (%)	Altered *n* (%)
VO_2_ VT1 (mL/kg-min)	<11 Altered		
	>11 Normal	15 (60)	10 (40)
VE/VCO_2_ (slope in degrees)	>36 Altered		
	<36 Normal	19 (76)	6 (24)
OUES (mL)	<1550 Altered		
	>1550 Normal	16 (64)	9 (36)
COP (L)	>30 Altered		
	<30 Normal	17 (68)	8 (32)
Dif. VO_2_/HR VT2-VT1	<0 Altered		
	>0 Normal	13 (52)	12 (48)

VO_2_/HR; and “altered” when over the cut-off point for VE/VCO_2_ and COP and the inverse situation for the “normal” condition. The other values presented in the table with cut-off points for altered and normal values are as follows: VT1 (First ventilatory threshold oxygen consumption); VE/VCO_2_ (ventilatory class, ventilation divided by carbon dioxide volume); OUES (Oxygen uptake efficiency slope); COP (Cardiorespiratory Optimal Point); diff. VO_2_/HR VT2-VT1 (oxygen pulse difference between VT2 and VT1).

**Table 4:** Normal (N) and altered (A) categorical scores, comparison between variables per Chi square. The numbers represent the condition (N or A), the number of subjects in this condition as the first number, followed by the percentage in parentheses, *p* value and * significant difference.

Score	Dif. VO_2_/HR		*p*-Value	VE/VCO_2_		*p*-Value	COP		*p*-Value	OUES		*p*-Value
	N	A		N	A		N	A		N	A	
VO_2_ VT1			0.87			0.57			0.28			0.17
N	8 (53.3)	7 (46.7)		12 (80.0)	3 (20.0)		9 (60.0)	6 (40.0)		8 (53.3)	7 (47.7)	
A	5 (50.0)	5 (50.0)		7 (70.0)	3 (30.0)		8 (80.0)	2 (20.0)		8 (80.0)	2 (20.0)	
Dif. VO_2_/HR	---	---	---			0.29			0.11			0.27
N	---	---		11 (84.6)	2 (15.4)		7 (3.8)	6 (46.2)		7 (53.8)	6 (46.2)	
A	---	---		8 (6.7)	4 (33.3)		10 (83.3)	2 (16.7)		9 (75.0)	3 (25.0)	
VE/CO_2_			0.29	---	---	---			0.94			0.04 *
N	11 (57.9)	8 (42.1)		---	---		13 (68.4)	6 (31.6)		10 (52.6)	9 (47.4)	
A	2 (33.3)	4 (66.7)		---	---		4 (66.7)	2 (33.3)		6 (100)	0	
COP			0.11			0.94	---	---	---			0.32
N	7 (41.2)	10 (58.8)		13 (76.5)	4 (23.5)		---	---		12 (70.6)	5 (29.4)	
A	6 (75.0)	2 (25.0)		6 (75.0)	2 (25.0)		---	---		4 (50.0)	4 (50.0)	
OUES			0.27			0.04 *			0.32	---	---	---
Normal	7 (43.8)	9 (56.2)		10 (62.5)	6 (37.5)		12 (75.0)	4 (25.0)		---	---	
Altered	6 (66.7)	3 (33.3)		9 (100)	0		5 (5.6)	4 (44.4)		---	---	

N: normal; A: altered. Normal means that it is above the cut-off point in the case of first-threshold oxygen consumption (VO_2_ VT1); Oxygen uptake efficiency slope (OUES); oxygen pulse difference (dif. VO_2_/HR between VT2-VT1) and low cut-off in ventilatory class, ventilation divided by carbon dioxide volume (VE/VCO_2_); Cardiorespiratory optimal point (COP). Altered is the opposite condition, i.e., under the cut-off point in VO_2_ VT1; OUES; DIF. VO_2_/HR between VT2-VT1; on the cut-off point of VE/VCO_2_ and COP.

## Data Availability

The datasets generated and/or analyzed during the current study are not publicity available due the terms of consent/assent to which the participants agreed but are available from the corresponding author on reasonable request. Please contact the corresponding author to discuss availability of data and materials.
